# Research Trends of Acupuncture Therapy on Polycystic Ovary Syndrome: A Bibliometric Analysis

**DOI:** 10.1155/2022/1989401

**Published:** 2022-10-12

**Authors:** Yiyi Wang, Yunfan Xia, Rongrong Li, Ruohan Sun, Jianqiao Fang

**Affiliations:** ^1^The Third Clinical Medical College, Zhejiang Chinese Medical University, Hangzhou 310000, China; ^2^The Third Affiliated Hospital, Zhejiang Chinese Medical University, Hangzhou 310000, China

## Abstract

**Background:**

Acupuncture has been confirmed as a suitable therapy for treating polycystic ovary syndrome (PCOS). However, there is no bibliometric analysis of the global use of acupuncture for PCOS. Our study used CiteSpace (5.8.R3) to provide a profile of the current state and trends in this field.

**Methods:**

Articles regarding acupuncture therapy for treating PCOS were retrieved from the Web of Science Core Collection. CiteSpace was used to analyze the number of publications, countries, institutions, journals, authors, cited references, and keywords by using standard bibliometric indicators.

**Results:**

A total of 159 publications were considered for the final analysis. The number of publications has slowly increased with fluctuations between years, and the most active countries, institutions, journals, and authors concerning acupuncture therapy for PCOS were identified. *Evidence-Based Complementary and Alternative Medicine* was the most productive journal, and Fertil Steril was the most cited. China and Heilongjiang University of Chinese Medicine were considered the most prolific countries and institutions in this field, respectively. Elisabet Stener Victorin became the most influential author and most cited author. Jedel E. published the most cited article. “Polycystic ovary syndrome” was the most frequent keyword, and the top three frontiers mentioned were research method, intervention, and outcome.

**Conclusion:**

The current status and trends in clinical research of acupuncture therapy on PCOS patients are revealed according to the results of this bibliometric study, which may facilitate researchers to identify hot topics and new directions for future study in this field.

## 1. Introduction

Polycystic ovary syndrome (PCOS) is one of the most common endocrine disorders among women of childbearing age. It is characterized by hyperandrogenism, ovulatory dysfunction, and polycystic ovarian morphologic features, usually with manifestations of irregular menses, infertility, hirsutism, and acne [1, 2]. Besides, patients with PCOS are generally burdened with insulin resistance, leading to a higher incidence of type 2 diabetes and cardiovascular and cerebrovascular diseases [3, 4]. The pathogenesis of PCOS has not been well defined concerning the multiple complicated factors, including genetic, environmental, and transgenerational components [5, 6]. Depending on different diagnostic criteria, the worldwide prevalence of PCOS ranges from 4% to 21%, 4%–6.6% according to NIH 1990 criteria, and 4%–21% by Rotterdam 2003 criteria [7]. This disease has severely affected the quality of patients' daily lives and significantly increased the odds of moderate to severe depression and anxiety [8, 9].

To date, there is no good treatment option to improve the symptoms of PCOS. Education, self-empowerment, multidisciplinary care, lifestyle intervention, and weight management are prioritized treatment options [10]. However, few studies have shown that they positively impact ovulation and live births despite their actual improvement in patients' weight, menstrual cycles, and emotions to a certain extent [11–13]. Besides, long-term adherence to treatments is challenging. As second-line therapy, pharmacological treatment always chooses combined oral contraceptives (COCs) to alleviate menstrual irregularity and hyperandrogenism, which have been proven to increase the risk of arterial thrombosis and venous thromboembolism [10, 14]. Meanwhile, letrozole, as the first-line pharmacological therapy for infertility, has been associated with musculoskeletal adverse events, vasomotor symptoms, vaginal dryness, hot flashes, and osteoporosis [15–17]. Furthermore, surgical procedures should only be used after all other treatment options have failed, as they will expose patients to surgery-related risks such as anesthetic difficulties, infection, and adhesions [6]. Apart from the interventions above, acupuncture, as a complementary and alternative therapy, has been confirmed as a suitable treatment for PCOS [18]. Clinical evidence shows that acupuncture has a specific effect on improving the symptoms of PCOS [19–22], but the treatment method and efficiency still need further research.

Bibliometrics is an interdisciplinary science that uses mathematical and statistical methods to analyze all knowledge carriers quantitatively. It is used to evaluate a specific discipline's social and scientific importance during a given period [23]. CiteSpace is an information visualization software jointly developed by Professor Chaomei Chen of Drexel University and the WISE Laboratory of the Dalian University of Technology. It can reveal hot spots and frontier directions in a particular field by visualizing the literature [24–26] and is widely applied in bibliometric analysis for its powerful functions and convenient operation.

As of yet, there has been no specific bibliometric analysis of the global use of acupuncture for PCOS, which presents an obstacle for researchers in obtaining a comprehensive understanding of the current status in this area. Therefore, it is necessary to conduct a bibliometric analysis, through which we can confirm the most influential countries, institutions, journals, and authors on acupuncture therapy for PCOS. This study aims to perform a macrolevel overview of the relevant academic literature through bibliometric analysis, thus evaluating the research trends of acupuncture treatment of PCOS, identifying the hot topics and frontiers, and paving the way for researchers to acquire relevant knowledge overall.

## 2. Materials and Methods

CiteSpace was utilized to do the bibliometric analysis of the current status and research trends of the global application of acupuncture for PCOS treatment. The literature eligible for retrieval was found in the Web of Science Core Collection.

All data of this study were retrieved from the Web of Science Core Collection on August 11, 2022, including Science Citation Index Expanded (2008-present), Current Chemical Reactions (1985-present), and Index Chemicus (1993-present). The search strategy included the topics “polycystic ovary syndrome” and “acupuncture therapy” without restrictions on the countries, categories, or languages. The literature published in 2022 was excluded from the analysis for incomplete results. The specific search strategies and results are shown in Table 1.

A bibliometric analysis was conducted on the identified publications through CiteSpace (5.8.R3) to reveal the annual output counts, prolific journals, authors, institutions, and countries, and explore the trends and patterns [27]. Meanwhile, we also investigated the collaborative relationships, such as co-occurrence analysis of institutions, authors, references, and keywords. The current research foundation, cutting-edge knowledge, and research trends of acupuncture treatment for PCOS were uncovered through bibliometric visualization.

CiteSpace was configured as follows: time slicing was done from 2008 to 2021, according to the search results, one year per slice (1); the term source was selected in its entirety; node types were selected one at a time; and the top 50 objects were applied in the selection criteria, and pathfinder was chosen in pruning. The visual knowledge figure was mainly composed of nodes and lines. Every node in the figure represented one element, such as country, institution, or author, and the bigger the node size, the higher the frequency of occurrence. Different colors suggest different years. From the interior to the outside nodes, the circles of various colors indicated the years 2008 to 2021. The importance of each node was roughly evaluated by the indicator of betweenness centrality (BC), which Freeman defined in 1977. A node with a high BC (≥0.1) was usually considered a pivotal point and marked with a purple circle. Moreover, lines between the nodes manifested cooperation, co-occurrence, or co-citation relationships [28–30].

## 3. Results

### 3.1. Annual Publications

**Figure 1:**
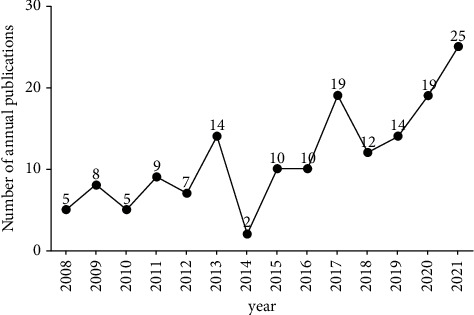
The annual number of publications on acupuncture therapy for PCOS.

A total of 175 publications were retrieved from the Web of Science Core Collection, including 111 records in articles, 48 records in review articles, 8 records in meeting abstracts, 6 records in letters, 1 record in correction, 1 record in book chapters, and 1 record in editorial material, according to the document types of the Web of Science Core Collection. Only records in articles and review articles, a total of 159 publications, were included in the analysis after removing duplicates [31].  Figure 1 depicts the number of specific articles published each year and reveals that the first paper on the application of acupuncture in treating PCOS was published in 2008. The figure indicated that the number of relevant publications had slowly increased, with some fluctuations. From 2008 to 2013, the number of publications increased from 5 to 14, despite reductions in 2010 and 2012. A plummet appeared in 2014 when the number was 2, the lowest in years, and then it was restored to 10 in 2015. Later, the number of published articles kept increasing, except for a shrink in 2018, and reached a peak of 25 in 2021.

### 3.2. Analysis of Journals and Cited Journals

**Figure 2:**
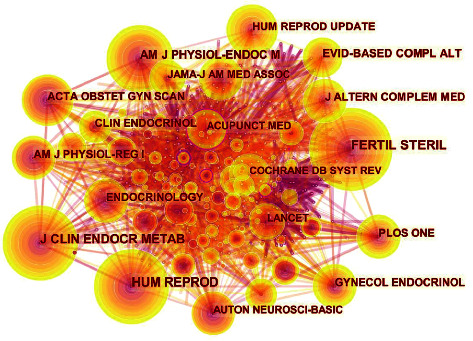
The cited journal map related to research of acupuncture treatment for PCOS. The nodes in the map represent journals, and links between the nodes signify cooperation relationships. The diverse colors of the nodes represent different years. The larger the node area, the greater the number of co-citations. The purple ring indicates the centrality of literature, and nodes with high centrality are considered pivotal points.

In total, 159 papers were published in 76 different journals. The top five journals on acupuncture treatment for PCOS are listed in Table 2. The average impact factor (IF) of the top 5 journals was 2.516. Evidence-Based Complementary and Alternative Medicine had the highest IF of 3.014, and was the most productive journal with 15 publications. CiteSpace generated a visualized map (Figure 2 and Table 3) of the cited journals based on 1699 references. The nodes in the map represented journals, and links between the nodes reflected co-citation relationships. Additionally, the purple-ringed nodes that emphasized the centrality of literature were usually regarded as the key locations. Hence, Fertil Steril ranked first in frequency, and Ann Intern Med had the highest centrality.

### 3.3. Distribution of Countries and Institutions

**Figure 3:**
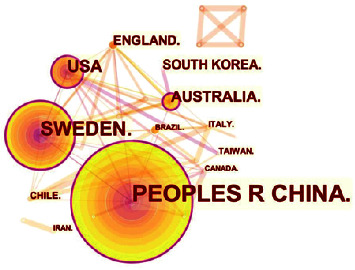
map of countries concerning research of acupuncture treatment for PCOS. The nodes in the map represent countries or territories, and links between the nodes signify cooperation relationships. The diverse colors of the nodes represent different years. The larger the node area, the greater the number of publications. The purple ring indicates the centrality of literature, and nodes with high centrality are considered pivotal points.

A map concerning countries' distribution was generated, composed of 26 nodes and 64 links, as shown in Figure 3. Researchers from 26 countries and regions wrote 159 articles on acupuncture therapy for PCOS. From Table 4, Sweden (0.20), the United States (0.19), China (0.17), Australia (0.11), and England (0.09) were identified as the top five nations in terms of centrality as well as the number of publications, regardless of the difference in rankings.

**Figure 4:**
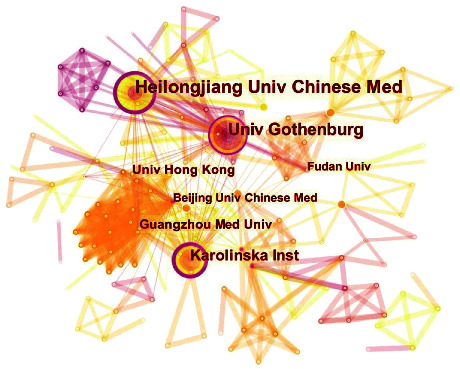
A map of institutions related to acupuncture treatment for PCOS. The nodes in the map represent institutions, and the links between the nodes signify collaborative relationships. The diverse colors of the nodes represent different years. The larger the node area, the greater the number of publications. The purple ring indicates the centrality of literature, and nodes with high centrality are considered pivotal points.

Figure 4 depicts a distribution map of institutions with 230 nodes and 685 lines. There were 230 institutions dedicated to the field of acupuncture therapy for PCOS, with Heilongjiang University of Chinese Medicine (35), University of Gothenburg (29), Karolinska Institute (20), University of Hong Kong (10), and Guangzhou Medical University (10) being the top five in terms of publication numbers (Table 5). Interestingly, Heilongjiang University of Chinese Medicine not only owned the highest publication numbers but also the greatest centrality of 0.25, equal to Karolinska Institute (0.25) and followed by Beijing University of Chinese Medicine (0.17), University of Gothenburg (0.16), and Peking University (0.15).

### 3.4. Analysis of Authors and Cited Authors

**Figure 5:**
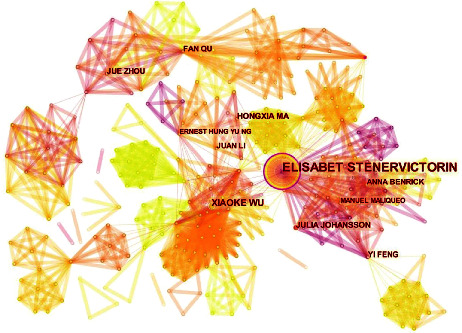
A map of authors dedicated to acupuncture treatment for PCOS. The nodes in the map represent authors, and the links between the nodes signify collaborative relationships. The diverse colors of the nodes represent different years. The larger the node area, the greater the number of publications. The purple ring indicates the centrality of literature, and nodes with high centrality are considered pivotal points.

The visualization map of authors aims to uncover the most productive author or co-author and reveal the cooperative relationship among them, which can provide valuable information regarding core research teams and potential collaborators and assist researchers in building new collaborative relationships [32]. The authors' map was generated after analyzing 159 publications, and 701 nodes and 2509 lines (Figure 5) were included. The 5 most published authors were Elisabet Stener Victorin, Xiaoke Wu, Julia Johansson, Hongxia Ma, and Anna Benrick, and Elisabet Stener Victorin, Rong Li, Fan Qu, Richard S. Legro, and Yi Feng had the top 5 centralities (Table 6). Elisabet Stener Victorin from the Karolinska Institute in Sweden was the most prolific author in terms of publications and centrality. A randomized controlled trial, performed by Elisabet Stener Victorin, on the impact of electroacupuncture (EA) and physical exercise on hyperandrogenism and oligo-amenorrhea in PCOS women found that low-frequency EA and physical exercise both significantly improved hyperandrogenism and menstrual frequency, and the effect of low-frequency EA was superior to physical exercise [33]. However, another randomized controlled trial of hers demonstrated that acupuncture, with or without clomiphene (the first-line treatment for infertility), did not increase live births of PCOS women in China compared with acupuncture and placebo in the control group [34]. Despite this conclusion, one of her reviews still believed that acupuncture was a recommendable treatment for women with PCOS, which could improve PCOS-related symptoms, including irregular menstruation, health-related quality of life, emotions, and insulin sensitivity with minimal side effects [35].

**Figure 6:**
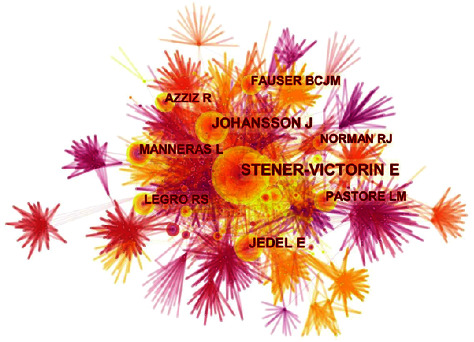
A map of cited authors dedicating to acupuncture treatment for PCOS. The nodes in the map represent co-cited authors, and links between the nodes signify co-citation relationships. The diverse colors of the nodes represent different years. The larger the node area, the greater the number of co-citations. The purple ring indicates the centrality of literature, and nodes with high centrality are considered pivotal points.

A map of the cited authors was produced, which consisted of 1705 nodes and 7553 lines (Figure 6). Interestingly, Elisabet Stener Victorin was the most cited author about acupuncture therapy for PCOS with 101 counts in total, followed by Johansson J (65), Jedel E (53), Manneras L (43), and Legro RS (38) (Table 7). As for centrality, the top 5 cited authors were Barber TM (0.19), Andersson S (0.12), Avis NE (0.12), Andersen CL (0.11), and Blank SK (0.10) (Table 7). Then, Barber TM, who had the highest centrality, served in the Oxford Centre for Diabetes, Endocrinology, and Metabolism (OCDEM), a world-class center for clinical research on diabetes, endocrine, and metabolic disorders, along with clinical treatment and education. Some of his reviews and studies explored the genetics, pathogenesis, and metabolic characteristics of PCOS, which were often referred to by related researchers as basic information [36–39].

### 3.5. Analysis of Cited References

**Figure 7:**
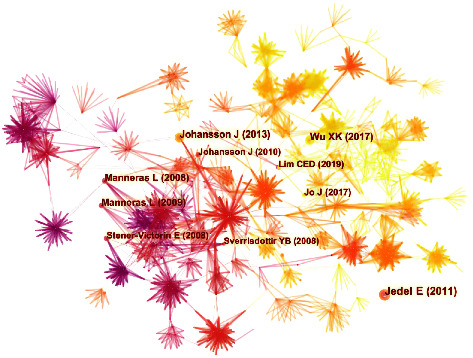
A map of cited references related to acupuncture treatment for PCOS. The nodes in the map represent cited references, and the links between the nodes signify co-citation relationships. The diverse colors of the nodes represent different years. The larger the node area, the greater the number of co-citations. The purple ring indicates the centrality of literature, and nodes with high centrality are considered pivotal points.

The map of cited references with 1699 nodes and 6229 links was acquired by analyzing 159 publications (Figure 7). The top 5 cited references in frequency and centrality are shown in Tables 8 and 9. The four of the top five most frequently cited references were all randomized controlled trials of acupuncture therapy for PCOS, three of which had been mentioned previously. The remaining article demonstrated that low-frequency EA and physical exercise could lower high sympathetic nerve activity, which was considered one of the causes of PCOS [40, 41]. Moreover, the review published by Lim CED in 2019, which ranked first in centrality, concluded that compared to sham acupuncture, receiving true acupuncture may improve the number of menstrual days of PCOS women [42]. The following publication, which was still a review of Lim's CED, suggested that acupuncture may have affected PCOS by increasing blood flow to the ovaries, reducing ovarian volume and number of ovarian cysts, improving insulin sensitivity, lowering cortisol levels, assisting in weight loss, etc [43].

### 3.6. Analysis of Keywords

**Figure 8:**
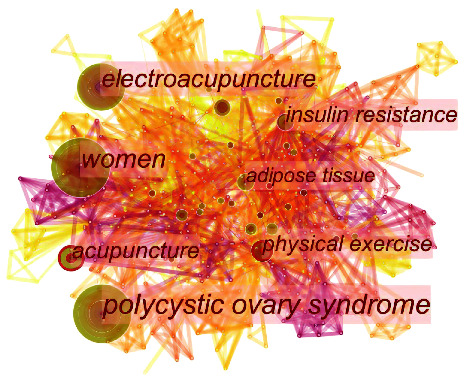
A map of keywords related to acupuncture treatment for PCOS. The nodes in the map represent keywords, and the links between the nodes signify co-occurrence relationships. The diverse colors of the nodes represent different years. The larger the node area, the greater the number of co-occurrences. The purple ring indicates the centrality of literature, and nodes with high centrality are considered pivotal points.

**Figure 9:**
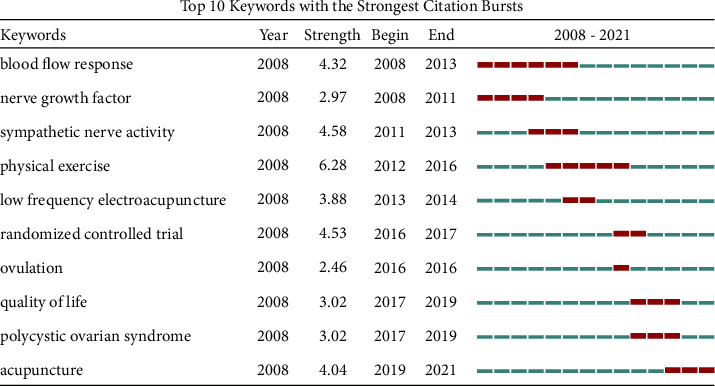
The top 10 keywords with the strongest citation bursts. The red bars indicate that the keyword was cited frequently; the green bars mean that the keyword was cited infrequently.

It was believed that keywords could reveal the research frontiers and emerging trends in a specific field with an increasing frequency of citations in a given period [44]. The keyword co-occurrence map was generated in Figure 8 with 598 nodes and 3159 links.  Table 10 shows the top 5 keywords with the highest frequency and centrality related to acupuncture therapy for PCOS. The most popular keywords included “polycystic ovary syndrome,” “women,” “electroacupuncture,” “acupuncture,” “insulin resistance,” and “adipose tissue.” Besides, the top 10 keywords with the strongest citation burst were identified by using burst detection (Figure 9). The most recent keyword bursts were “acupuncture,” “polycystic ovary syndrome,” “quality of life,” “ovulation,” and “randomized controlled trial,” which indicated that the three frontiers in burst strength were research method, intervention, and outcome.

## 4. Discussion

According to the above results, the status of acupuncture therapy on PCOS can be exposed as follows.

The fluctuant growth in the number of articles suggests that acupuncture, as a supplementary and alternative medicine, has received increasing attention in recent years. More studies have been done to determine its effectiveness in PCOS treatment. However, the amount of literature is limited overall, and no sign of rapid growth.

Evidence-Based Complementary and Alternative Medicine is the most productive journal, with the highest IF of 3.014, indicating its maximum impact in the area. Furthermore, the average IF of 2.516 in the top five journals indicates limited attention to the field. Fertil Steril broadly influences infertility and human reproductive disorders, while Ann Intern Med possesses authority in the field.

China ranks first in the aspect of publications with a total of 96 references, probably because China is the origin of acupuncture. As the second-ranked country, Sweden has 42 references, indicating the country's broad application of acupuncture treatment for PCOS. While the Heilongjiang University of Chinese Medicine, the most prolific and critical institution, may own the most influential position in this field. Besides, Karolinska Institute and the University of Gothenburg are in the top five in terms of publications and centrality and are located in Sweden. Thus, it can be assumed that Chinese and Swedish institutions give more priority to the research on acupuncture therapy for PCOS.

Elisabet Stener Victorin, the most productive and cited author, undoubtedly holds substantial influence in the field of acupuncture treatment on PCOS, and the effectiveness of electroacupuncture in improving PCOS-related symptoms is one of her research directions. Besides, Julia Johansson and Richard S Legro are among the top five productive and cited authors, indicating they are active researchers and excellent potential collaborators in the field. Regrettably, there is little clinical research regarding acupuncture treatment on PCOS among the publications of the influential cited authors, which indicates a dearth of high-quality clinical evidence to confirm the effectiveness of acupuncture in the treatment of PCOS.

According to the top five cited references, it can be considered that the comprehensive analysis of acupuncture treatment for PCOS is mainly concerned with randomized controlled trial, review, and endocrine metabolic-based regulatory mechanism. Nevertheless, the limited number of randomized controlled trial and the lack of high-quality clinical evidence have made it challenging to accurately evaluate acupuncture therapy's effectiveness for PCOS.

As the most important and popular research method of evaluating the efficacy of acupuncture therapy on PCOS, randomized controlled trial can provide high-quality evidence. The keyword “acupuncture,” emerging from 2019 and possessing the strength of citation burst of 4.04, suggests that acupuncture therapy has gained increasing attention in recent years. And the keyword “low-frequency electroacupuncture,” which existed from 2013 to 2014 with the strength of citation burst of 3.88, indicates that low-frequency electroacupuncture is the most commonly applied acupuncture therapy in the treatment of PCOS. Furthermore, more relevant randomized controlled trials have been conducted to evaluate the effects of acupuncture on improving the quality of life, ovulation rates, and insulin resistance of PCOS patients [22, 45, 46]. Regrettably, there is a lack of convincing evidence for the efficacy of acupuncture in treating women with PCOS [47]. Therefore, more well-designed randomized controlled trials should be carried out for further investigation.

In addition, our research had several drawbacks. First and foremost, because Web of Science was the only database we used to search for relevant publications, the results of our study were inherently incomplete. As a result, additional analysis of articles in other databases, particularly some Chinese databases, is critical. Besides, although the research topic has been defined during searching, there is no guarantee that all retrieved literature is totally relevant to the issue. Despite this, we believe this study retains some credibility in describing the overall state and potential prospects in this field.

## 5. Conclusions

Acupuncture has been proven to be a good treatment for PCOS as a supplementary and alternative therapy and has the advantages of clinical efficacy and few side effects. Much of the relevant literature in the study was published in Evidence-Based Complementary and Alternative Medicine journals. China was the most productive country in this area, and the Heilongjiang University of Chinese Medicine was the most prolific institution. While Elisabet Stener Victorin of the Karolinska Institute in Sweden was named the most influential author and the most cited author simultaneously, which implied that acupuncture therapy was increasingly accepted worldwide. Additionally, randomized controlled trials were commonly carried out to explore the efficacy of acupuncture treatment on PCOS, and low-frequency electroacupuncture was the most popular acupuncture therapy. The primary outcome measures were quality of life, ovulation rates, and insulin sensitivity. Nevertheless, the limited number of randomized controlled trials and a lack of high-quality clinical evidence constrained researchers from evaluating the effectiveness of acupuncture therapy for PCOS. Thus, more well-designed randomized controlled trials should be conducted for further investigation.

Overall, this study first fills the gap of bibliometric analysis on acupuncture therapy for PCOS and then reveals the present state and trends in this field, which may help researchers identify hot topics and explore new research paths.

## Figures and Tables

**Table 1:** The topic search query.

Set	Results	Search query
^#^1	15143	(TS = (acupuncture OR acupuncture therapy OR acupuncture treatment OR acupuncture treatments OR body acupuncture OR needle acupuncture OR manual acupuncture OR warm acupuncture OR electroacupuncture OR electroacupuncture)) Indexes = Web of Science Core Collection, Timespan = 1985–2021

^#^2	16127	(TS = (PCOS OR polycystic ovary syndrome OR polycystic ovarian syndrome)) Indexes = Web of Science Core Collection, Timespan = 1985–2021

^#^3	175	^#^1 AND ^#^2

**Table 2:** Top 5 scholarly journals related to acupuncture therapy on PCOS.

Rank	Publications	Journal	IF (Five years)
1	15	Evidence-Based Complementary and Alternative Medicine	3.014
2	10	Medicine	2.227
3	10	Acupuncture in medicine	2.192
4	8	Chinese Journal of Integrative Medicine	2.395
5	6	Trials	2.754

**Table 3:** Top 5 cited journals and centrality related to acupuncture therapy on PCOS.

Rank	Frequency	Cited journal	Rank	Centrality	Cited journal
1	126	Fertil Steril	1	0.32	Ann Intern Med
2	112	Hum Reprod	2	0.13	Acupunct Med
3	100	J Clin Endocr Metab	3	0.12	Am J Public Health
4	90	Am J Physiol-Endoc M	4	0.11	Brit Med J
5	72	Evid-Based Compl Alt	5	0.10	Chin J Integr Med

**Table 4:** Top 5 publications and centrality of countries related to acupuncture therapy on PCOS.

Rank	Publications	Country	Rank	Centrality	Country
1	96	Peoples R China	1	0.20	Sweden
2	42	Sweden	2	0.19	USA
3	24	USA	3	0.17	Peoples R China
4	16	Australia	4	0.11	Australia
5	10	England	5	0.09	England

**Table 5:** Top 5 publications and centrality of institutions related to acupuncture therapy on PCOS.

Rank	Publications	Institution	Rank	Centrality	Institution
1	35	Heilongjiang Univ. Chinese Med	1	0.25	Heilongjiang Univ. Chinese Med
2	29	Univ. Gothenburg	2	0.25	Karolinska Inst.
3	20	Karolinska Inst.	3	0.17	Beijing Univ. Chinese Med
4	10	Univ. Hong Kong	4	0.16	Univ. Gothenburg
5	10	Guangzhou Med Univ.	5	0.15	Peking Univ.

**Table 6:** Top 5 publications and centrality of authors related to acupuncture therapy on PCOS.

Rank	Publications	Author	Rank	Centrality	Author
1	38	Elisabet Stener Victorin	1	0.18	Elisabet Stener Victorin
2	13	Xiaoke Wu	2	0.10	Rong Li
3	9	Julia Johansson	3	0.07	Fan Qu
4	9	Hongxia Ma	4	0.06	Richard S Legro
5	8	Anna Benrick	5	0.03	Yi Feng

**Table 7:** Top 5 frequency and centrality of cited authors related to acupuncture therapy on PCOS.

Rank	Frequency	Cited author	Rank	Centrality	Cited author
1	101	Stener Victorin E	1	0.19	Barber TM
2	65	Johansson J	2	0.12	Andersson S
3	53	Jedel E	3	0.12	Avis NE
4	43	Manneras L	4	0.11	Andersen CL
5	38	Legro RS	5	0.10	Blank SK

**Table 8:** Top 5 frequency of cited references related to acupuncture therapy on PCOS.

Rank	Frequency	Cited reference	Author and publication year
1	32	Impact of electroacupuncture and physical exercise on hyperandrogenism and oligo/amenorrhea in women with polycystic ovary syndrome: a randomized controlled trial	Jedel (2011)
2	25	Effect of acupuncture and clomiphene in Chinese women with polycystic ovary syndrome: a randomized clinical trial	Wu (2017)
3	23	Acupuncture for ovulation induction in polycystic ovary syndrome: a randomized controlled trial	Johansson (2013)
4	21	Low-frequency electroacupuncture and physical exercise decrease high muscle sympathetic nerve activity in polycystic ovary syndrome	Stener Victorin (2009)
5	20	Acupuncture and exercise restore adipose tissue expression of sympathetic markers and improve ovarian morphology in rats with dihydrotestosterone-induced PCOS	Manneras (2009)

**Table 9:** Top 5 centrality of cited references related to acupuncture therapy on PCOS.

Rank	Centrality	Cited reference	Author and publication year
1	0.13	Acupuncture for polycystic ovarian syndrome	Lim (2019)
2	0.12	Current evidence of acupuncture on polycystic ovarian syndrome	Lim (2010)
3	0.11	Longitudinal antimüllerian hormone in women with polycystic ovary syndrome: an acupuncture randomized clinical trial	Franasiak (2012)
4	0.11	Anxiety and depression in polycystic ovary syndrome: a comprehensive investigation	Deeks (2010)
5	0.10	Reproductive and metabolic phenotype of a mouse model of PCOS	Leonie (2012)

**Table 10:** Top 5 frequency and centrality of cited authors related to acupuncture therapy on PCOS.

Rank	Frequency	Keyword	Rank	Centrality	Keyword
1	82	Polycystic ovary syndrome	1	0.25	Insulin resistance
2	56	Women	2	0.21	Electroacupuncture
3	45	Electroacupuncture	3	0.19	Acupuncture
4	35	Acupuncture	4	0.17	Adipose tissue
5	32	Insulin resistance	5	0.15	Women

## Data Availability

The raw data can be directly obtained from the Web of Science Core Collection (WoSCC).
